# Evaluation of two evidence-based decision aids for female *BRCA1/2* mutation carriers in Germany: study protocol for a randomised controlled parallel-group trial

**DOI:** 10.1186/s13063-022-06081-7

**Published:** 2022-02-16

**Authors:** Sibylle Kautz-Freimuth, Marcus Redaèlli, Anna Isselhard, Arim Shukri, Andrea Vodermaier, Kerstin Rhiem, Rita Schmutzler, Stephanie Stock

**Affiliations:** 1 Institute of Health Economics and Clinical Epidemiology, The University Hospital of Cologne, Gleueler Straße 176-178, 50935 Cologne, Germany; 2 School of Population and Public Health, The University of British Columbia, 2206 East Mall, Vancouver, BC C6T 1Z3 Canada; 3 Centre for Familial Breast and Ovarian Cancer, Centre for Integrated Oncology (CIO), Faculty of Medicine and The University Hospital of Cologne, University of Cologne, Kerpener Straße 62, 50937 Cologne, Germany

**Keywords:** *BRCA1* mutation, *BRCA2* mutation, Decision aid, Decision-making, Evaluation study, Familial breast and ovarian cancer, Hereditary breast and ovarian cancer (HBOC), Preventive measures, Preference-sensitive decisions, Patient-centred care

## Abstract

**Background:**

Women with *BRCA1/2* mutations have a higher risk of developing breast and ovarian cancer compared to women of the general population. Various preventive options are available to deal with the increased risk of developing cancer. These include intensified breast cancer screening and risk-reducing bilateral mastectomy and salpingo-oophorectomy. The choice of a preventive option can lead to increased decisional conflict. To support these women in their decision-making process, two evidence-based decision aids were developed in an upstream research process and adapted to the German healthcare context. These will be evaluated within a randomised controlled trial (RCT) in terms of their effects on decision-making, women’s level of information and psychological outcome variables.

**Methods:**

A sample of 310 women carrying *BRCA1/2* mutations (A) without a history of cancer or (B) with a history of unilateral breast cancer who have received post-test genetic counselling will be enrolled. Upon study consent, women will be randomly assigned to either the intervention or the control group. All participants will receive standard care including a physician’s letter summarising the counselling content. After baseline data collection (t0), the intervention group receives the respective decision aid while the control group receives standard care only. The primary outcome variable assessed at a 3-month follow-up (t1) is the change of extent in decisional conflict (measured with the Decisional Conflict Scale). Secondary outcome variables comprise the stage of decision-making, self-reported symptoms of anxiety, depression and stress due to the genetic test result, and knowledge regarding cancer risks and preventive options. At t1, the extent of preparation for decision-making and acceptability of the decision aids will also be examined. Another secondary outcome variable assessed at 6-month follow-up (t2) is the extent of decision regret.

**Discussion:**

These will be the first decision aids available for *BRCA1/2* mutation carriers in Germany to be evaluated regarding their effectiveness and acceptability in clinical use within an RCT. Subsequently, they are to be integrated into the care concept of the centres of the German Consortium for Hereditary Breast and Ovarian Cancer and the affiliated breast centres.

**Trial registration {2a}:**

DRKS DRKS00015823. Retrospectively registered on 14 June 2019

## Administrative information

Note: The numbers in curly brackets in this protocol refer to SPIRIT checklist item numbers. The order of the items has been modified to group similar items (see http://www.equator-network.org/reporting-guidelines/spirit-2013-statement-defining-standard-protocol-items-for-clinical-trials/).

**Table:** 

**Title {1}**	Evaluation of two evidence-based decision aids for female *BRCA1/2* mutation carriers in Germany: study protocol for a randomised controlled parallel-group trial
**Trial registration {2a, 2b}**	DRKS-ID: DRKS00015823. Registered 14 June 2019 - Retrospectively registered, http://www.drks.de/drks_web/navigate.do?navigationId=trial.HTML&TRIAL_ID=DRKS00015823.
**Protocol version {3}**	Study protocol version No. 1 [23/12/2020]
**Funding {4}**	This trial is funded by the Landeszentrum Gesundheit Nordrhein-Westfalen (LZG.NRW), Gesundheitscampus 10, 44801 Bochum, Germany.
**Authors and Affiliations {5a}**	Kautz-Freimuth S^1^, Redaèlli M^1^, Isselhard A^1^, Shukri A^1^, Vodermaier A^1,3^, Rhiem K^2^, Schmutzler R^2^, Stock S^1^ Affiliations ^1^ Institute of Health Economics and Clinical Epidemiology, The University Hospital of Cologne, Gleueler Straße 176-178, 50935 Cologne, Germany. ^2^ Centre for Familial Breast and Ovarian Cancer, Centre for Integrated Oncology (CIO), The University Hospital of Cologne, Kerpener Straße 62, 50937 Cologne, Germany. ^3^School of Population and Public Health, The University of British Columbia, 2206 East Mall, Vancouver, BC C6T 1Z3, Canada.
**Name and Contact Information for the Trial Sponsor {5b}**	The University Hospital of Cologne, Kerpener Straße 62, 50937 Cologne, Germany.
**Role of Study Sponsor and Funder {5c}**	Neither the study sponsor nor the funder is involved in the study design, data collection, data management, data analyses and interpretation, report writing, decision to submit this report for publication or the writing of this publication.

## Introduction

### Background and rationale {6a}

Women with a pathogenic germline mutation in the *BRCA1* or *BRCA2* gene face a high risk of developing breast cancer (BC) and ovarian cancer (OC). The average cumulative life-time risk for BC increases with age [1, 2], reaching about 70% by the age of 80 [1]. The average cumulative lifetime risk for OC is around 44% (*BRCA1* mutation) and 17% (*BRCA2* mutation) [1]. Compared to women affected by sporadic BC or OC, *BRCA1/2* mutation carriers without a history of cancer, in the following referred to as ‘previvors’ [3], develop BC or OC about 20 years earlier in their life. Those with a history of unilateral BC, in the following referred to as ‘survivors’ [4], face an average cumulative 20- to 25-year risk of contralateral BC of about 40 to 44% (*BRCA1* mutation) or around 26 to 33.5% (*BRCA2* mutation) [1, 5].

Newly diagnosed *BRCA1/2* mutation carriers are offered various preventive options to counter their increased cancer risks. These include an intensified breast cancer screening programme for previvors or an intensified breast cancer screening and aftercare programme for survivors and risk-reducing surgeries of the breasts and the adnexa for both groups. Intensified breast cancer screening (breast magnetic resonance imaging (MRI), breast ultrasound and mammography) enables BC to be detected at an early, potentially curable stage in 85% of cases [6], but does not reduce the risk of developing BC. Women who opt for screening in the first place can postpone their final decision to have surgery. Due to the limited specificity of the MRI, screening often yields false-positive results [6] which can lead to further often more invasive diagnostic tests (e.g. re-imaging or breast biopsies) which may later prove unnecessary and may trigger transient anxiety in women [7]. In contrast, risk-reducing bilateral mastectomy significantly decreases the risk of developing BC [8] for previvors and provides a survival benefit to *BRCA1* mutation carriers [9]. Risk-reducing contralateral mastectomy lowers the risk of contralateral BC and reduces overall mortality in survivors [10]. However, removal of the breasts is an irreversible decision that affects physical integrity and requires further decisions, e.g. which form of surgery or whether and, if so, which breast reconstruction the woman would prefer. For survivors, the decision-making process may be even more complex because they may face competing risks (e.g. risk of BC recurrence on the affected side) that may have to be weighed against the benefits of risk-reducing surgery on the non-affected side.

In the absence of an effective screening method for the adnexa [11–14], the only preventive option to counter the risk of OC is risk-reducing bilateral salpingo-oophorectomy. It reduces both the risk of OC [15] and the overall and OC specific mortality [16]. However, consequences include the definite loss of fertility and possible premature menopause; the latter can cause menopausal symptoms such as hot flashes, as well as long-term consequences such as cardiovascular disease and osteoporosis [17].

Each preventive option is accompanied with distinct advantages and disadvantages that each mutation carrier will judge and weigh individually depending on her personal experiences, values and preferences. The same applies to the several options of breast reconstructions after mastectomy, to family planning or to steps to be taken to treat undesired effects of an option [18]. For example, to counter the negative consequences of surgical menopause after a risk-reducing bilateral salpingo-oophorectomy, temporary hormone replacement therapy might be considered for premenopausal women [11]. Hence, *BRCA1/2* mutation carriers face several so-called preference-sensitive decisions [19, 20]. These can lead to considerable decisional conflicts that can be associated with delays of decisions, dissatisfaction, decision regret or blaming of healthcare providers [21–26]. These negative consequences might be further complicated by lacking knowledge and understanding of the individual risk constellation and available options or by personal stressors and psychosocial, family and/or psychological factors [20, 26–29].

In order to support *BRCA1/2* mutation carriers during their complex decision-making process in choosing a preventive option and the right time to do so, a number of supportive tools, in particular, decision aids (DAs), have been developed internationally [30–36]. A systematic review on DAs for women with *BRCA1/2* mutations identified four RCTs and one pretest-post-test study that assessed the effectiveness of DAs on decision-, knowledge- and health-related criteria. The analysis showed that DAs support these women most likely by improving decision-related outcomes: Women who received a DA experienced lower decisional conflict were more likely to come to a decision and were more satisfied with the decision made compared to women who did not receive a DA [37]. Favourable effects of DAs on decision-related factors as well as on knowledge are reported by a previous Cochrane Review that analysed 105 RCTs involving a total of 31,043 participants with regard to the effects of DAs on patients facing treatment or screening decisions across different indications. High-quality evidence was found for the following effects: DAs improve knowledge about the available options, lower decisional conflicts resulting from the feeling of not being informed and support clarification of values and preferences of the addressees [38].

In Germany, following receipt of the genetic test result, women with a pathogenic *BRCA1/2* mutation receive personalised counselling from a medical specialist at one of the specialised GC-HBOC centres or their affiliated breast centres. The post-test genetic counselling and care concept (in the following referred to as ‘standard care’) includes detailed non-directive information on the women’s mutation status, their individual risk prediction, risks and benefits of the available risk-adapted prevention options and their consequences [11, 39–41], provision of written information, e.g. on self-help or psychological support options, along with a physician’s letter summarising the contents of the consultation. So far, no additional structured intervention is used to provide women carrying *BRCA1/2* mutations with targeted support for making high-quality decisions defined as being informed by the best available scientific knowledge and based on the women’s values and preferences [42, 43].

For this reason, two evidence-based DAs (one for previvors (DA-A), one for survivors (DA-B) with *BRCA1/2* mutations) that correspond to the evidence-based guidelines and consented procedures in the German healthcare system were developed in an upstream research process [44]. The development of the DAs followed a structured, quality-controlled procedure according to the International Patient Decision Aid Standards Collaboration [45–47]. Both DAs follow the same structure and share the same content for all aspects that are valid for both target groups (e.g. information on genetic mutations, methods of breast cancer screening). Yet, they differ in aspects where the respective target group needs and/or wishes distinct information. For example, previvors need information on the risk-reducing bilateral removal of healthy breasts, while survivors need information on BC in the affected breast and on the risk-reducing removal of the non-affected breast. Before the newly developed DAs can be incorporated into standard care, it is required to evaluate these in terms of their effectiveness and acceptability in clinical use [45, 48]. In order to fulfil this final quality criterion, the DAs will be evaluated in the randomised controlled trial (RCT) described here in detail.

### Objectives {7}

The aim of this study is to evaluate two newly developed evidence-based DAs for women with pathogenic *BRCA1/2* mutations in Germany with regard to their effectiveness on decision-, psychological- and knowledge-related factors in clinical use and their acceptability with the addressees. We hypothesise that the use of these DAs on top of standard care will reduce the extent of decisional conflict regarding the choice of a preventive option compared to standard care alone (primary outcome). Furthermore, it will be examined whether the use of the DAs can reduce possible symptoms of anxiety, depression or psychological strain associated with the genetic test result, the risk of BC and OC and the available preventive options. Lastly, we hypothesise that at 6-month-follow-up, the intervention group (IG) will feel less regret regarding the choice made compared to the control group (CG).

### Trial design {8}

The trial is designed as a monocentric randomised controlled parallel-group superiority trial with a 1:1 allocation ratio.

## Methods

The study protocol is based on the Standard Protocol: Recommendations For Intervention Trials (SPIRIT 2013 Statement) [49].

### Study setting {9}

All participants will be recruited by the medical specialist team supported by a study nurse (recruiting team) at the Centre for Familial Breast and Ovarian Cancer at the University Hospital of Cologne (recruiting institution). All data will be collected and analysed by the research team consisting of physicians, healthcare researchers, and a statistician at the Institute for Health Economics and Clinical Epidemiology at the University Hospital of Cologne (data evaluation institution).

### Eligibility criteria {10}

All participants must meet the inclusion criteria for genetic testing according to the German Consortium for Hereditary Breast and Ovarian Cancer (GC-HBOC) [50] and have undergone a genetic testing. Included in the study are women with a positive genetic test result for a pathogenic *BRCA1*/2 mutation who have received post-test genetic counselling and have not yet made a final decision on at least one preventive measure. This includes both mutation carriers immediately after the post-test genetic counselling and those later on after post-test genetic counselling who have initially decided to participate in the intensified breast cancer screening (and aftercare) programme but do not yet know, when and for which alternative they will finally opt.

Further inclusion criteria are as follows:

- (A) Not affected by cancer or (B) affected by unilateral BC (stage I, II or III)
- Age 18 to 70 years
- No medical reasons against possible risk-reducing surgeries
- Given written consent to participate in the study
- Sufficient knowledge of the German language

Exclusion criteria are as follows:

- Affected by advanced BC, e.g. local recurrence or distant metastasis
- Affected by ovarian cancer or other types of cancer other than unilateral BC
- Age under 18 years and over 70 years
- Medical reasons against possible risk-reducing surgery
- Not able to give informed consent/not given informed consent to participate in the study
- Insufficient knowledge of the German language

### Consent to participate {26a}

The recruiting physicians will obtain the informed written consent of potential trial participants after they have been informed about the study and are willing to participate. Informed consent will be obtained from all study participants.

### Additional consent provisions {26b}

In the consent form, the participants will also be asked to agree that in the event of withdrawal from the study, their data collected up to that point may be processed. No further consent provisions will be requested.

### Interventions

#### Choice of comparators {6b}

The participants of the CG will receive standard care. Standard care was chosen as the comparator, because it is offered for all women with pathogenic *BRCA1/2* mutations seeking advice by the specialised GC-HBOC centres as part of post-test genetic counselling and is currently considered the gold standard.

Standard care in the GC-HBOC centres is as follows: In a medical non-directive consultation mutation, carriers receive detailed individual information on their genetic test results, their lifetime, age- and/or time-dependent risks of BC and OC and the risk-adapted preventive options available to them. In addition, further written information may be provided, e.g. on the German self-help organisation BRCA network (BRCA-Netzwerk e.V.) or on psychological counselling options. Following a detailed physician’s consultation, each woman receives a personal physician’s letter summarising the contents of her consultation by regular mail. This includes information about the identified mutation, the calculated individual risks for breast and ovarian cancer, the intensified breast cancer screening programme with pros and cons, the risk-reducing surgeries of the breast and ovaries and fallopian tubes with pros and cons, possible further cancer risks, the significance of the genetic test result for a possible desire to have children, the probability of passing on the mutation to the offspring and a summary of the result of the counselling interview.

#### Intervention {11a}

Participants in the IG will also receive standard care as described above. After the return of the baseline questionnaire (t0), participants of the IG will be sent the DAs as a brochure by regular mail. Participants of the CG will not receive a DA.

#### Criteria for discontinuing or modifying allocated interventions {11b}

No known unfavourable side effects of the implementation of DAs are reported in the literature. Nevertheless, all participants will be offered to set up an appointment with the clinical psychologist at the recruiting institution in case stressful feelings or thoughts will be coming up. They will also be offered an additional specialist appointment at the centre if the need to clarify further issues arises. Participants will be informed that participation is completely voluntary and that they can leave the study at any time without giving any reason or having to fear negative consequences.

#### Strategies to improve adherence to intervention protocols {11c}

Participants of the IG are encouraged to read through the DAs and engage with them by filling out the attached work sheets and discuss its contents with persons of their confidence. No special strategy was defined to facilitate the use of the DAs. To support physicians’ assistance to recruit eligible participants into the study, a study nurse will be involved. The study nurse will review the patient files with upcoming appointments every day and will place the enrolment documents in the files of eligible women.

#### Concomitant care or interventions {11d}

Not applicable. No concomitant care or interventions are planned. No concomitant care or interventions are explicitly prohibited.

### Outcomes {12}

Baseline measures at t0 will be obtained within 2 weeks after enrolment in the study, t1 measures 3 months and t2 measures 6 months after enrolment (see Table 1). At baseline (t0), the following variables will be collected: decisional conflict as measured with the Decisional Conflict Scale (DCS) [51], stage of decision-making as measured with the Stage of Decision Making Scale, SDM-S [52], anxiety and depressive symptoms as measured with the Hospital Anxiety and Depression Scale, HADS [53, 54], impact of genetic test result as measured with the Impact of Event Scale-Revised, IES-R [55] and knowledge criteria as measured with a set of fifteen knowledge questions.

**Table 1:**
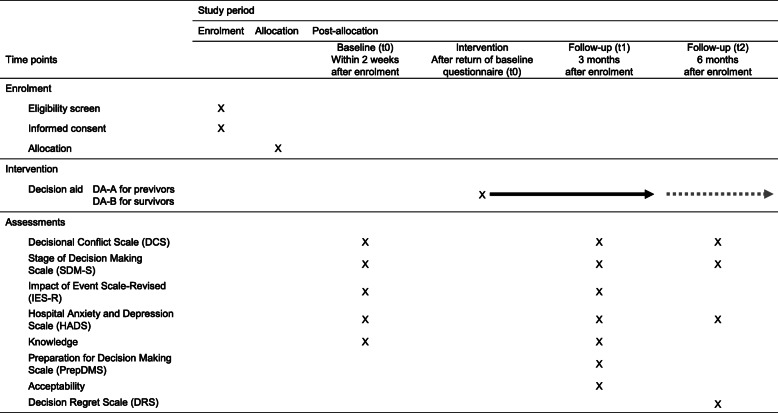
Outcome parameters and time points for outcome measurements

The primary outcome at 3-month follow-up (t1) is the change in the extent of decisional conflict measured with the DCS. Secondary outcome variables at t1 include changes in SDM-S, HADS, IES-R and knowledge. Additionally, at t1, the items addressing the preparation for decision-making as measured with the Preparation for Decision Making Scale (PrepDMS) [56, 57] and the acceptability of the DAs compared to control by the participants as measured with an acceptability instrument [30, 58] are collected. Secondary outcome variables at t2 comprise SDM-S, DCS, HADS and the extent of regret for the decision made as measured with the Decision Regret Scale (DRS) [59]. It is possible that no final decision has been made at t2 or that the decisions made at t2 are only temporary.

The evaluation instruments are described below.

#### Decisional conflict

Decisional conflict is measured using the Decisional Conflict Scale (DCS) [51, 60]. It comprises five subscales with a total of 16 items, which are assessed on a 5-point Likert scale (from 1 = strongly agree to 5 = strongly disagree). The subscales concern the following topics: being informed, clarification of personal values, support or pressure from others, uncertainty about the decision and the assessment of one’s own decision-making. The German version of the DCS has been shown to have good psychometric properties, with a reported internal consistency of Cronbach’s alpha = 0.78 or higher [60]. The DCS is administered at t0, t1 and t2.

#### Stage of decision-making

The stage of decision-making is measured using the Stage of Decision Making Scale (SDM-S) [52]. It consists of a single item with four or six response categories. In this study, an adapted German version with a four-answer category is used [61]. The categories used are (1) ‘I have not yet thought about the options’, (2) ‘I am considering the options’, (3) ‘I am close to choosing one option’ and (4) ‘I have already made a choice’. The SDM-S is administered at t0, t1 and t2.

#### Anxiety and depressive symptoms

To measure the symptoms of anxiety and depression, the German version of the Hospital Anxiety and Depression Scale (HADS-D) [53, 54] is used. It measures the level of anxiety and depressive symptoms in the last week by self-assessment. The HADS-D consists of two subscales (anxiety, depression) with seven items each, which are assessed on a 4-point Likert scale. The tool is widely used and shows good psychometric properties, with most studies reporting an internal consistency of Cronbach’s alpha = 0.8 or higher. It has been shown to be well suited for measuring emotional stress in cancer patients [62]. The HADS-D is administered at t0, t1 and t2.

#### Subjective stress symptoms because of the genetic test result

To determine the impact of the genetic test result of having a pathogenic *BRCA1/2* mutation on subjective stress symptoms the German version of the Impact of Event Scale-Revised (IES-R) is used [63]. The IES-R contains a total of 22 items in three subscales referred to as intrusion, avoidance and hyperarousal. They are assessed using a 4-point Likert scale (from 0 = not at all to 5 = often). The values of the three subscales can be used to calculate the probability of diagnosing a post-traumatic stress disorder. The IES-R is administered at t0 and t1.

#### Knowledge on cancer risks and preventive options

Knowledge about cancer risks associated with the *BRCA1/2* mutations and the available preventive options conveyed by the present DAs will be tested with a set of fifteen statements that can be classified as ‘true’, ‘not true’ or ‘don’t know’. In preparation for this study, this instrument was developed by a team of medical experts, psychologists and healthcare researchers working in the field of hereditary breast and ovarian cancer (HBOC). The topics covered by this instrument are addressed in the DAs and distributed as follows: four items each deal with the risks of BC/OC and risk-reducing breast surgery, three items each deal with the intensified breast cancer screening (and aftercare) programme and with risk-reducing adnexal surgery. One item concerns a topic that is explained in a section addressing ‘questions and answers’ of the DAs. Women’s level of knowledge is assessed at t0 and t1.

#### Preparation for decision-making

The German version of the Preparation for Decision Making Scale (PrepDMS) [57, 64, 65] will be used to measure the extent to which the participants feel prepared for the decision by the additional DA. The instrument consists of the two subscales ‘preparation for the decision’ and ‘preparation for the physician’s consultation’ and comprises a total of ten items, which are rated on a 5-point Likert scale (from 1 = not at all to 5 = a great deal). This instrument is validated, shows good internal consistency (Cronbach’s alpha = 0.95 for total score) and is recommended for the evaluation of DAs [65]. The PrepDMS is administered at t1.

#### Acceptability

Acceptability of the DAs among participants of the IG is measured by an acceptability scale. Participants of the CG will judge the written information received as part of standard care with the same instrument. The tool is adapted from an acceptability tool developed by O’Connor and Cranney [58] and from an approach to test acceptability described in a study by Metcalfe et al. [30]. It consists of seven items in which personal assessments of the following characteristics of the received DA are queried: (1) scope, (2) amount of information, (3) comprehensibility, (4) usefulness in making a decision on a preventive measure, (5) satisfaction with the DA, (6) sufficient information to make an appropriate decision and (7) likelihood of recommending the DA to other women in her situation. Items (1), (2), (6) and (7) are assessed with a dichotomous response format; items (3), (4) and (5) are scored with a 3-step ordinal response format. The acceptability scale is administered at t1.

#### Decision regret

To assess the participants’ level of distress and regret in terms of the final decision made, the Decision Regret Scale (DRS) [21, 59, 66] is used. It consists of five items to be rated on a 5-point Likert scale (from 1 = strongly agree to 5= strongly disagree). The instrument was validated in a sample of BC patients and showed good internal consistency with a Cronbach’s alpha = 0.81 to 0.92. It is strongly negatively correlated with decision satisfaction and overall quality of life and positively correlated with decisional conflicts [21]. The DRS is administered at t2.

The outcome parameters and time points for outcome measurements are listed in Table 1.

### Participant timeline {13}

**Fig. 1:**
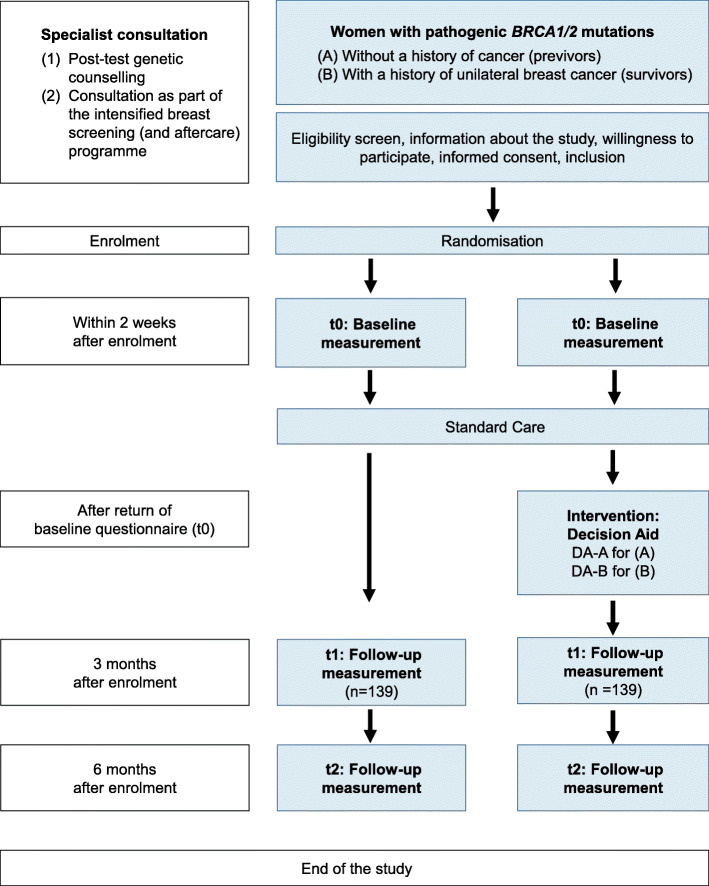
Participant timeline

The schematic participant timeline is shown in Fig. 1.

### Enrolment and randomisation

Recruitment takes place during a specialist consultation either (1) directly following the post-test genetic counselling or (2) during a medical examination as part of the intensified breast cancer screening programme (for previvors, A) or the intensified breast cancer screening and aftercare programme (for survivors, B). If the inclusion criteria are met, eligible women will be informed about the study and invited to participate. Women who agree to do so will be asked to provide informed consent. Physicians will complete a recruitment form. Thereafter, participants are randomly assigned to one of the two study groups. The allocation is blinded to both the recruiting physicians and the participants.

### Baseline data collection at t0

After enrolment, all participants receive a package with study material (enrolment package). It contains the baseline questionnaire (t0) with a pre-stamped return envelope, brief and detailed information about the study and a copy of the informed consent document. Participants are invited to fill out the questionnaire at home and to return it within 2 weeks per regular mail.

### Intervention

Once the completed baseline questionnaires (t0) have been returned to the data evaluation institution, the DAs will be sent by regular mail to those participants randomised to the IG with a request to use the DA for information and decision support at home. Previvors will receive DA-A, and survivors will receive DA-B.

### Outcome data collections at t1 and t2

Participants, who have returned the baseline questionnaire (t0), will receive the follow-up questionnaire at t1 at 3-month-follow-up with the request to return the completed questionnaire within two weeks by regular mail. Participants who have returned the questionnaire t1 will receive the next follow-up questionnaire at t2 at 6-month follow-up with the request to return the completed questionnaire within two weeks by regular mail.

### Sample size {14}

The required sample size was calculated based on the effect size (Cohen’s *d*) from previous research [30, 67, 68]. Since the effect size varies between 0.3 and 0.83, a conservative assumption is made with a small effect size of 0.3, *α* of 0.05, and *ß* of 0.8. Since numerous studies provide evidence that DAs significantly reduce decisional conflict [30, 38, 69], a superiority study with a one-sided *t-*test is conducted. Using the one-sided *t-*test, a sample size of 139 patients per group is required. Assuming an average drop-out rate of 10% of *BRCA1/2* mutation carriers in previous evaluation studies [31, 32, 34, 69, 70], the planned sample size is *n* = 155 participants per group, yielding a total of 310 required participants.

### Recruitment {15}

The following strategies are used to achieve adequate participant enrolment to reach the target sample size: Posters are placed in the waiting room areas of the recruitment centre to publicise the study and invite interested women to participate. A study nurse will check women’s files who have upcoming appointments in the recruiting institution and screen these for eligibility. Then, counselling physicians are requested to actively approach eligible women after post-test genetic counselling or during an appointment for the intensified breast cancer screening (and aftercare) programme.

#### Allocation: sequence generation {16a}

Study participants will be randomly assigned to either IG or CG with a 1:1 allocation. Sequence generation is done with a computerised random number generator by a member of the research team who is not involved in data analysis.

#### Allocation concealment mechanism {16b}

After sequence generation, a member of the research team prepares the enrolment packages that contain the study documents for eligible participants. There are no differences in the enrolment packages between IG and CG. Thus, all packages look exactly the same and contain the same documents. Each package is marked with a study ID. Only the members of the research team at the data evaluation institution know the allocation of the study IDs to IG and CG. The participants, the recruiting physicians and the study nurse will not be able to decode the allocation.

#### Implementation {16c}

The enrolment packages are prepared and equipped with a study ID by the data evaluation institution and then transferred to the recruitment institution. After informed consent, the recruiting physicians enrol eligible women into the study, and each participant receives an enrolment package. As the allocation to IG or CG is determined by the study ID, both physicians and participants are blinded regarding the assignment of the participants to IG or CG.

#### Blinding {17a}

Once the completed baseline questionnaire (t0) is returned to the data evaluation institution, the research team will identify study IDs that are assigned to the IG and prepare the sending of the DA in a closed envelope. This envelope will be solely marked with the study ID. An independent entity neither belonging to the research team nor the recruiting team will decode the study ID, prepare the envelope with the name and address of the participants and send it off. The supervising study nurse remains blinded to the allocation to the study group throughout the study. This also applies to the recruiting physicians. However, it cannot be ruled out that treating physicians could be unblinded if participants approach them about the study at a follow-up appointment. At t0, all trial participants are blinded. The data analysts will be blinded with regard to group allocation by coding group allocation before data analysis.

#### Unblinding {17b}

Due to the nature of this study, unblinding for the participants will occur as soon as they receive the DAs. The same applies to participants who receive the follow-up questionnaires at t1 and have not received a DA by then. With the exception of the statistician who is responsible for data analysis, the research team will be able to assign the evaluated data to the two study groups and therefore will not be blinded.

### Data collection, management and analysis

#### Data collection methods {18a}

The completed questionnaires are sent to the data evaluation institution, where the research team collects the data and transfers them into a digital form on a password-protected computer. The research team is neither involved in post-test genetic counselling nor in recruitment of the participants.

#### Promoting participant retention and complete follow-up {18b}

Participants who did not return the questionnaires in time will receive up to two postal reminders with the request to send the questionnaires back within 1 week. Those who still will not react will be contacted again by telephone to encourage participant retention. This will be done by the study nurse who works with the recruiting team.

Women assigned to the CG do not receive DAs and may, therefore, be more likely to drop out of the study than women assigned to the IG. To counter this possibility of higher drop-out rates, all women in the CG are offered to request a DA when the last follow-up questionnaire at t2 is returned. This is to ensure that they can participate in the study without being disadvantaged.

#### Data management {19}

The paper-based data from the returned pseudonymised questionnaires will be transferred into digital data using the software programme Remark Office OMR. For quality control and verification, all the extracted data is also manually checked by members of the research team. According to the German data protection guidelines, the paper-based questionnaires are kept in a securely locked location, as are the digital data which is stored on a notebook with password protection and kept in a securely locked location. Solely, the members of the research team have access to these data.

#### Confidentiality {27}

According to the German data protection guidelines, personal information about potential and enrolled participants is kept strictly separate from the study data in order to maintain confidentiality before, during and after the trial. Personal data is only known to the recruiting physicians, the study nurse and one independent person who is responsible for sending the DAs to the IG participants (see the ‘Blinding {17a}’ section). Only these persons will have a list that allows the study IDs to be matched to the personal data of the participants. This assignment list will be kept strictly separate from the study data collected by the research team. The research team will never have access to the participants’ personal data but will only receive pseudonymised questionnaires.

#### Biological specimens {33}

No biological specimen will be collected.

## Statistical methods

### Analysing primary and secondary outcomes {20a}

Analysis of the baseline data (t0) will be done to ensure the comparability of the two study groups. Outcomes are measured at t1 and t2. All primary and secondary effectiveness variables will be described by statistical characteristics values. Continuous data will be described by mean, standard deviation, median, minimum and maximum. Categorical data will be described by using frequencies and percentages. The number of non-missing values will also be given.

The primary outcome at t1 is decisional conflict. The mean differences of the primary outcome between both groups will be compared using the independent *t-*test in case of normal distribution. In addition, the mean differences in the score within the groups between t0 and t1 will be compared using a dependent *t-*test. Non-parametric tests will be used in case of non-normal distribution. Secondary outcomes comprise the mean differences in scores of the stage of decision-making, stress, anxiety and depressive symptoms between t0, t1 and t2. A further secondary outcome measured at t2 is the regret of the decision. For these analyses, a dependent respectively independent *t-*test will be used in case of normal distribution, non-parametric tests will be used in case of non-normal distribution. Data will be analysed by using IBM SPSS Statistics for Windows, version 27.0 (IBM Corp: Armonk, NY) and R [71]. An alpha level of 0.05 is considered significant in all statistical tests.

### Interim analysis {21b}

An exploratory interim analysis of the baseline data collected at t0 is planned when a total of 70 participants have been recruited. The aim is to check whether the baseline data of both study groups are comparable. Access to the analysed interim data will be given to the project leading member (SKF) and the statistician who conducts data analysis (AS).

### Additional analyses {20b}

Subgroup analyses are planned to investigate whether there are differences in primary and secondary outcomes (1) between *BRCA1/2* mutation carriers without a personal history of cancer and those with a personal history of unilateral BC and (2) between women recruited directly after the post-test genetic counselling or later on at an appointment as part of an examination for the intensified breast cancer screening (and aftercare) programme. Subgroup analyses will be performed using the independent *t-*test for normally distributed metric variables, and non-parametric tests will be used in case of non-normal distributions. The chi-square test or Fisher’s exact test will be used for categorical variables. Beyond group allocation, demographic data will be used as the independent variable.

### Definition of analysis population {20c}

All analyses will be conducted following the intention-to-treat (ITT) principle including all randomised patients. No imputation of missing values will be performed.

### Public access {31c}

The datasets that will be generated and/or analysed during the present study will be available from the corresponding author on reasonable request.

### Roles and responsibilities {5d}

The coordination team includes two scientific staff members and the medical director. Their tasks are to prepare the ethics application, produce the study materials, train the clinical recruitment units, prepare the reports for the funding institution, coordinate the consulting experts and obtain the consent forms. In addition to the coordination team, the steering committee includes the management of the participating institutions. It reviews progress, discusses interface problems and proposes adjustments if necessary. Data management is organised by two study nurses and two research assistants who work spatially and structurally separate from the coordination team. They are divided into two units. One takes over pseudonymisation, dispatch and reminder functions. The other performs data entry, plausibility checks and data extraction, and delivers the data set for data evaluation.

### Monitoring

#### Data monitoring {21a}

Data monitoring is carried out by members of the research team, which consists of healthcare researchers, physicians and a statistician. The monitoring is performed completely independently of the funding institution. None of the members of the data monitoring committee has competing interests. No independent data monitoring committee has been installed, as it is not expected that any adverse events or interim analyses will lead to a recommendation to terminate the study prematurely.

#### Plans for collecting adverse events {22}

The use of the DAs is not expected to have significant undesired effects on participants. Therefore, no specific plan for the collection, assessment, reporting and managing of possible spontaneous unintended effects is provided and no formal guideline for termination of use will be established.

#### Auditing {23}

Trial conduct will be audited every 6 months by the scientific staff members working within the project. A semi-annual reporting will be sent to the funding institution (LZG.NRW). Further data monitoring is carried out by another scientist who can conduct quality assurance audits at any time. The competent supervisory institutions, e.g. the responsible ethics committee, have the right to inspect the study documents and reports or the data management processes at any time, while maintaining confidentiality.

#### Protocol amendments {25}

The Ethics Committee of the Medical Faculty of the University of Cologne will be notified of amendments to the protocol with the application for approval. The changes will also be communicated to the funding institution. Amendments will not be implemented before the final ethical approval is given.

#### Dissemination policy {31a}

The trial results are intended to be published in scientific journals.

## Discussion

This study is the first evaluation trial in which two DAs are examined that were specifically developed for female *BRCA1/2* mutation carriers in Germany. The study will be conducted within an RCT design. The overall objective is to further enhance the current specialised care and counselling concept for these women by providing quality and effectiveness tested decision support tools that potentially aid the decision-making process and thus may help to reduce decisional conflict. Secondary outcomes address the questions of whether both DAs can also improve the level of knowledge, positively affect psychological strain symptoms, are well accepted by the addressees, and may help to reduce regret regarding the final decision.

Both DAs target *BRCA1/2* mutation carriers have the same structure, contain the same elements and are identical in many aspects of content, but additionally, DA-A addresses specific needs of previvors, while DA-B addresses specific needs of survivors. Nevertheless, both DAs can be evaluated in a single RCT, because the research question refers to DAs’ impact on the decision-making process rather than to the options the women eventually will choose.

The procedures in the study have some limitations which, however, cannot be avoided due to the nature of this type of intervention chosen. One limitation is that there is no guarantee that all IG participants will actually use the DAs which they will receive per regular mail and are invited to use at home. In order to comply with the voluntary nature of the study participation and to avoid exerting any pressure on study participants, the follow-up questionnaire at t1 which collects data after receipt of the DA does not ask whether the DA was actually used. Therefore, the research team will receive no feedback as to whether and if so, to what extent the women have worked with the DA. On the other hand, discussions with the members of the BRCA network and with medical specialists who provide genetic counselling indicated that many women with *BRCA1/2* mutations hope for a device in which all aspects of their decision-making process are explained and brought together. A further limitation refers to the possibility that study participants might get in contact with each other. For example, due to the hereditary nature of the gene mutation, multiple family members may take part in the study. In this case, women assigned to the CG or women at baseline could unintentionally have access to the DA. It will also remain unclear, to what extent the study participants will use other sources of information or support. Such interferences may contaminate the collected data to a certain extent. Yet, there is no way to prevent this and accessing additional information is assumed to happen equally in the IG and CG. Another limitation refers to the blinding of participants, which is not possible throughout the entire course of the present trial. Since all participants will be informed in detail about the course of the study before inclusion into the study, they all are aware that they may or may not receive the intervention. Blinding of the participants can therefore only be guaranteed at baseline (t0). For IG participants, unblinding occurs at the moment they receive the DAs. CG participants are unblinded at the latest when they receive the follow-up questionnaire at t1 and have not yet received the DAs. It seems most appropriate to examine the impact of both DAs in a setting that reflects the current concept of standard care. This implies that the CG participants do not receive other information than that provided by the current concept. Finally, it cannot be excluded that there may be a bias due to non-response by not returning questionnaires, especially if non-response rates should differ between IG and CG.

This real life-based comparison of IG versus CG is, therefore, also a strength of this study. Another convincing strength is the study design. An RCT offers a high degree of quality control for both DAs and enables results of high significance for clinical care. Comparing standard care with standard care plus, DA allows for more clarity on what kind of support *BRCA1/2* mutation carriers seeking advice can expect from the use of DAs in clinical practice. Furthermore, valuable information for revisions of both DAs may be provided by study participants.

Following the evaluation described in the present study protocol, the DAs both for previvors and survivors will be integrated into the care and counselling concept of the GC-HBOC centres and their affiliated breast centres. This is intended to make an important contribution to strengthening the decision-making competence and autonomy of women with *BRCA1/2* mutations.

## Trial status

Study protocol version No. 1.0 [23/12/2020]. The recruitment of the study participants started in January 2019. The recruitment closed on 30 September 2021.

## Abbreviations

BC: Breast cancer
*BRCA1/2*: BReast CAncer genes 1 and 2
CG: Control group
DA-A: Decision aid A (designed for previvors)
DA-B: Decision aid B (designed for survivors)
DAs: Decision aids
DCS: Decisional Conflict Scale
DRS: Decision Regret Scale
GC-HBOC: German Consortium of Hereditary Breast and Ovarian Cancer
HADS: Hospital Anxiety and Depression Scale
ID: Identity number
IES-R: Impact of Event Scale-Revised
IG: Intervention group
MRI: Magnetic resonance imaging
RCT: Randomised controlled study
OC: Ovarian cancer
PrepDMS: Preparation for Decision Making Scale
SDM-S: Status of Decision Making Scale
